# Sensors Energy Optimization for Renewable Energy-Based WBANs on Sporadic Elder Movements

**DOI:** 10.3390/s22155654

**Published:** 2022-07-28

**Authors:** Anand Singh Rajawat, S. B. Goyal, Pardeep Bedi, Chaman Verma, Calin Ovidiu Safirescu, Traian Candin Mihaltan

**Affiliations:** 1 School of Computer Sciences and Engineering, Sandip University, Nashik 422213, India; anandsingh.rajawat@sandipuniversity.edu.in; 2 Faculty of Information Technology, City University, Petaling Jaya 46100, Malaysia; 3 Department of Computer Science & Engineering, Galgotias University, Greater Noida 203201, India; pradeep.bedi@galgotiasuniversity.edu.in; 4 Department of Media and Educational Informatics, Faculty of Informatics, Eotvos Lorand University, 1053 Budapest, Hungary; chaman@inf.elte.hu; 5 Environment Protection Department, Faculty of Agriculture, University of Agriculture Sciences and Veterrnary Medicine Cluj-Napoca, Calea Manastur No. 3-5, 400372 Cluj-Napoca, Romania; 6 Faculty of Building Services Engineering, Technical University of Cluj-Napoca, 400114 Cluj-Napoca, Romania; mihaltantraian83@gmail.com

**Keywords:** Boltzmann machines, sensor nodes, sporadic elder movements, wireless body area network

## Abstract

The world is advancing to a new era where a new concept is emerging that deals with “wirelessness”. As we know, renewable energy is the future, and this research studied the integration of both fields that results in a futuristic, powerful, and advanced model of wireless body area networks. Every new emerging technology does have some cons; in this case the issue would be the usage of excess energy by the sensors of the model. Our research is focused on solving this excessive usage of energy to promote the optimization of energy. This research work is aimed to design a power-saving protocol (PSP) for wireless body area networks (WBANs) in electronic health monitoring (EHM). Our proposed power-saving protocol (PSP) supports the early detection of suspicious signs or sporadic elder movements. The protocol focuses on solving the excessive energy consumption by the body attached to IoT devices to maximize the power efficiency (EE) of WBAN. In a WSNs network, the number of sensor nodes (SNs) interact with an aggregator and are equipped with energy harvesting capabilities. The energy optimization for the wireless sensor networks is a vital step and the methodology is completely based on renewable energy resources. Our proposed power-saving protocol is based on AI and DNN architectures with a hidden Markov model to obtain the top and bottom limits of the SN sources and a less computationally challenging suboptimal elucidation. The research also addressed many critical technical problems, such as sensor node hardware configuration and energy conservation. The study performed the simulation using the OMNET++ environment and represent through results the source rate to power critical SNs improves WBAN’s scheme performance in terms of power efficiency of Sporadic Elder Movements (SEM) during various daily operations.

## 1. Introduction

WBAN’s electronic health (eHealth) management system facilitates the integration of patient data collection and communication with healthcare resources or devices and is a potential method for improving healthcare quality. Sensor nodes in WBAN track the condition of the patients and transfer the summarized data to the doctor [1]. As a result of this study [2], a comprehensive approach to developing a maturity model was needed. That will include a wide variety of factors that concern healthcare facilities in all sectors and subsystems. In this paper [3], the author has represented the life of medical WBANs by using the relationship between disease recognition and applying the scheduling algorithm on the medical sensors. Xu et al. [4] suggests an energetic, effective distributed adaptive cooperative routing (EDACR) to WMSN following quotas and power usage restrictions as a disclosure of the energy efficiencies issue as a discretionary and finite-state decision-making mechanism (DFMDP). Reference [5] finds that WBANs face several matters of operations, standardization, and protection that concern consumer protection and privacy efficiency and maintenance. In reference [6], the proposed technology scans the sensors at near-optimum positions at various times, using the shortest path algorithm, to route information from target to destination. The wireless charging algorithm [7] proposed for WBAN implanted SNs is designed to be multi-collision prevention (CSMA/CA) per carrier sense access process. We used fusion protocols with improved performance parameters to create an energy-efficient [8] and stable WBAN link in this chapter. We demonstrated that durability while maintaining a low SAR level. Since a powerful network application necessitates a large number of network resources, CW and back-off mechanism adjustments in the MAC improve network capacity, PDR, energy consumption, and life span in various parametric combinations. Similarly, the latency and routing overheads for different parameter combinations show significant sleep variations. Cooperation with the proposed methods increased overall network reliability, as shown by the discussion of simulation outcomes, even though maximizing all performance parameters simultaneously is difficult in WBAN [9] performance enhancement. Instead, the WBAN implementation option takes priority over essential output parameters [10] (Sleep Mode, Network lifetime, Path loss, Stability, Residual energy, Packet delivery ratio). Therefore, various low-layer approaches can be added to promote improved network performance [11] and upper-layer protocols based on the sensitivity of the WBAN program. The methods employed in this study generally offered connection efficiency, stability and enhanced various network capacity parameters significantly [12]. Strengthening training focused on the Deep Neural Network [13]. The approach to improving learning is first to sensitize the radio environment and decide on a channel’s fate. No mathematical channel model has been established by IEEE 802.15.6, the IEEE standard paper [14], however, contains experimented values that are the basis for such a model. Based on these experimental findings, we developed a mathematical model that considers the posture of the body and the shadowing results. The WBAN sensors based on WSN sensors consist of a limited number of sensor nodes which work on low and unstable power energy. Several networks use parameters like communication, data storage, and the ability to perform several processes and sensing data. The WBAN sensors are capable of working on solar energy and wind energy, which are renewable energy resources. The WSN sensor working is completely based on low energy power systems, therefore the energy generated is unstable. Because several sensors run continually, renewable energy resources can be used to generate a continuous flow of energy. We suggest a new energy allocation algorithm to achieve the optimal degree of interference at the gateway boundary. Its primary condition for convergence is to minimize interaction with other gateways. Most of the research is conducted on renewable energy applications and on its usage, but this paper would cover the optimization of the sensors in order to optimize the model so that the renewable energy could be used optimally. The paper is based on the concept that whether the energy is in abundance or not, the model should be optimal for energy usage and productivity.

To organize the research work as a following manners: Section 2—related work done by the different number of researchers in WBANs. Section 3—Power consumption aspect in WBAN, sleep mode, Section 4—AI-based DNN architectures for WBANs on sporadic elder movements, Section 5—Composition of the WSN, Section 6—Transmission reliability, Section 7 outlines our simulation scenario as well as the detailed system model and Section 8—Results analysis and discussion introduces and addresses the simulation’s results, Section 9—Conclusion and future work. The research is focused on the problems that might arise after testing the model in a real environment. One of the major problems would be the issue of usage of the excess energy by the sensors. Although the energy is renewable energy and is plentiful, the ideal model still has some characteristics and one of them is to work efficiently with the least energy possible. In the research, we will show how the excess energy consumption can be detected and possible solutions for the energy optimization for the sensors.

## 2. Related Work

Joint pain and several bone-related problems were observed among older adults. The Wireless Body Area Networks (WBAN) sensors play an essential role in detecting older adults’ movements [15]. The WBAN sensors are wireless sensors capable of detecting physical parameters and many security concerns are associated [16]. The malicious data, unauthorized access, and DoS attacks are the most common attacks found in WBAN sensors [17]. The WBAN sensors are widely used in health monitoring and sports facilities to monitor several health parameters [18]. The deep learning techniques are used for detection and assessing the health data extracted using WBAN sensors. Deep Belief Network (DBN), Boltzmann Machine, and Deep Neural Network are used to detect various physical parameters associated with elderly people [19]. Additionally, several other parameters such as heart rate and breath rate can be detected Using WBAN sensors [20].

Table 1 primarily studies and shows the research methodology of some of the useful research used for this paper in brief. The table describes not just the summary and achievement of the citied paper but the research gaps as well.

Table 2 follows an analytical approach towards the citied papers which are directly related to the idea of our research. It shows the various approaches of different research and what their main objectives were.

## 3. Power Consumption Aspect in WBAN

**Figure 1:**
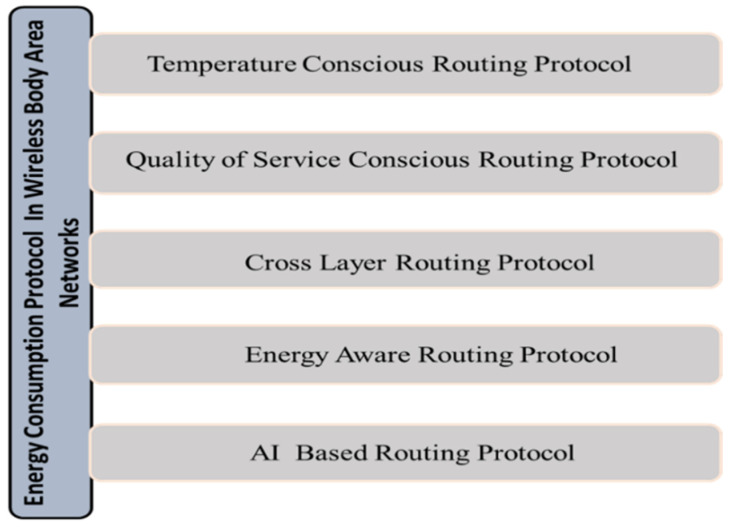
Routing protocol of WBAN.

The WBAN nodes should consume as little power as possible in order to extend the network’s lifespan and improve its stability. As the WBAN would use renewable energy, we must understand why we need to add the energy optimization feature in the model. The energy might be abundant, but while marking a model as ideal, we check some standards. For example, out of the given models which gives the most efficient output while being energy conservative. Such models are best for the longer run and promote energy conservation, model stability and energy optimization. In our case, the sensors might be one of the components which could be using energy a lot more than they actually need to or possibly the energy is not optimally used by the sensors and so the study addresses the uses and suggests some possible solutions. Using energy-efficient routing algorithms, we sought to reduce the WBAN’s power usage as much as possible in this section of the paper. Due to inherited difficulties and specifications, existing routing protocols for wireless sensor networks and mobile ad hoc networks do not work efficiently with WBAN. Figure 1 shows the WBAN routing protocols that are now in use, followed by a review of the various routing protocols that have been established so far.

When using the Quality of Service Conscious (QSC) Routing Protocol, a modular strategy-based protocol can increase parameters like dependability and packet delivery ratio [31]. These interconnected modules provide various challenges to the WBAN system’s design. In WBAN, biomedical sensors that detect changes in body temperature are placed inside or on the patient. Wireless communication generates magnetic and electric fields through radio transmissions. Absorption, power consumption, and the circuitry of the node all contribute to an increase in temperature when radio signals are present [32]. Consequently, the human body is vulnerable to the effects of these electromagnetic waves. In order to minimize the effects of the magnetic and electric fields generated by sensors, several approaches [33] have been developed. The use of computer clusters as the basis for the routing protocol (CBRP) nodes in this third type of routing protocol technique are used to form small clusters, and each cluster is led by a single cluster head. The only means to communicate with the base station is through this cluster head. This has resulted in a reduction in the amount of communication between sensors and base stations. Routing based on postural movement-based routing protocol (PMBRP) Postural movement introduces discontinuity issues into any relationship between two sensor nodes. Data packets are transmitted among sensor nodes with the least amount of postural movement possible. Routing protocols for data across several organizational layers [34], Network and MAC layer issues can be solved via cross-layer protocols, which improve the network as a whole. There is little doubt that energy-aware protocols, which prioritize node selection and mobility over the network’s energy or power consumption, are directly linked to the average lifetime and stability of the network. Routing Protocol Based on AI Crucial data takes precedence over less-important information in the analysis. Because a relay node in a routing process has no restriction on the amount of energy it may consume, emergency or critical data is prioritized over normal traffic because it must be delivered to its destination first and because it is necessary to monitor crucial medical information. Real-world energy components can be used to model this constraint:

PCO threshold

The composition of the energy in the model must be changed when we look at the working period of sensors and relays. Each cycle’s active and sleep time can be referred to as Power Cycle Optimization (PCO).

PCO = PSO (Activation condition) + PCO (Sleep condition) + PCO (switching condition)

PSO (Activation condition) = power utilization if the node is active condition

PCO (Sleep condition) = power utilization if the node is sleep condition

PCO (switching condition) = power utilization if the node is switching condition (active to sleep and sleep to active)

To performing the precise formulation

PCO = X PSO (Activation condition) +Y PCO (Sleep condition) + Z PCO (switching condition)

(1) $$\mathrm{PCO}=\overset{n}{\underset{i=1}{\sum }}{\mathrm{PCO}}_{{\mathrm{cycle}}_{i}}$$

We’ll calculate the energy consumption of correctly transmitted packets and the lifetime of the network, just like we did in the previous study. For any connection to effectively transmit a packet, the amount of energy consumed by PCO_ij_ is:

(2) $${\mathrm{PCO}}_{\mathrm{ij},x}=\frac{{\mathrm{PCO}}_{T\;X}^{Z}}{{\mathrm{PDR}}_{\mathrm{ij}}}$$

In this case, PDR_ij_ specifies the probability that a packet will be transported from I to j, while PCO Z TX indicates the energy needed to transmit the packet at z dBm, as stated. This is for when you only have to jump once. Node K, which serves as a relay between the sender I and receiver (j) in the two-hop transmission scenario:

(3) $${\mathrm{PCO}}_{\mathrm{ij}\;}^{K}=\frac{2\times {\mathrm{PCO}}_{T\;X}^{k}}{{\mathrm{PDR}}_{i}^{k}j}$$

A homogeneous wireless body area network would use the same TX power level for all sensors, but a heterogeneous wireless body area network would use a variable TX power level for each sensor (WBAN). When dealing with more complex situations, it is necessary to make additional modifications to the framework.

### Sleep Mode

Listening to idles is an essential factor in wasting energy, as nodes need to remain alive to receive possible results. If there is no information from the other transmitters, since a sleep node [35] is not working, no data can be obtained and no information broadcast. A successful Sleep Mode mechanism can bring resilience to energy conservation into the right balance. The core ideas for Body MAC’s [36] sleep mode are founded by the findings that specific nodes in WBAN are very duty-free and The receiving of the beacons in each frame and being involved on both the up and downlink is a waste of energy. The BodyMAC sleep mode attempts to disable the node radio as much as possible during beacon, uplink, and download. The sleep parameters in terms of frame number are the frame number and sleep time. Following the request from Sleep Mode, the node gateway sends an ACK to show that the demand for Sleep Mode was successfully received. The gateway determines the mode of sleep and sleeps criteria based on evidence or experience. This reveals that the Global Telecommunication System (GTS) data packet transfer loss means sleep mode termination. The re-sync follows the re-sync process. The supporting of time crucial incident monitoring is one of the specifications of the WBAN MAC [37] specification. The Sleep Mode must help it because time-critical events will still occur. If the node in the current frame is assigned to GTS [38], the event message packet may be sent to CAP [39] or GTS, in the nearest CAP time the incident report will be sent by CSMA/CA to the gateway [40]. When an ACK is received which indicates that an event report packet has been received successfully through the gateway, the node is reentered in sleep mode. GTS is another way to submit a packet of incident reports.

**Figure 2:**
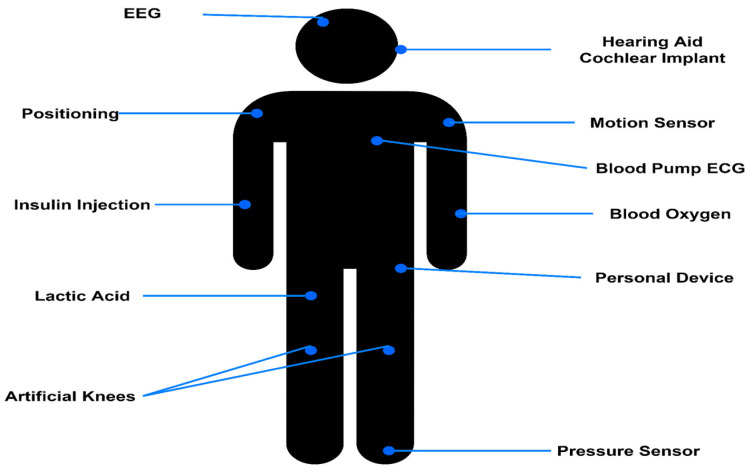
IoT Device.

WBASN connectivity architecture, the connectivity architecture of the WBASN monitoring system, is depicted in Table 3 and Figure 2. As the Figure 2 shows, the sensor attached human body. Electroencephalograms (EEGs) [41], electrocardiograms (ECGs), electromyography’s (EMGs), and blood pressure monitors are all examples of sensors. A dish or base station was used to transmit the sensor data to the central server (BS). A medical doctor, a medical clinic, or a medical library may then use this information to acquire additional information and diagnose a disease.

**Figure 3:**
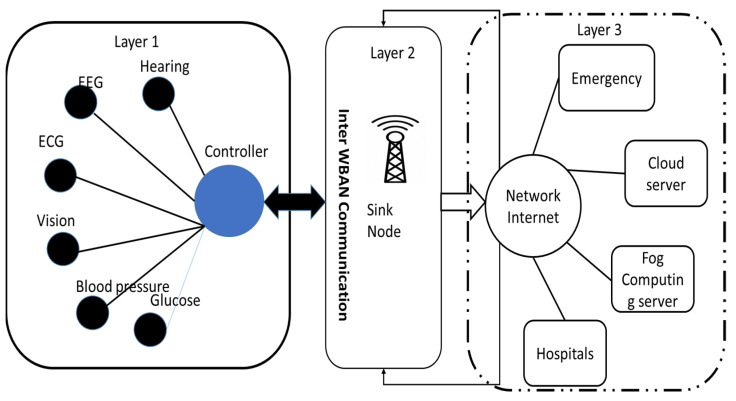
WBASN communication architecture.

Figure 3 represents the WBASN communication architecture. The hybrid system means combination of more modes of generation of electricity together, usually by means of renewable energy like that solar photovoltaic and wind power generators. Hybrid renewable systems offer a greater level of power security all the way through the combine of production, and over and over again will include a storage power system (power batteries, fuel cells) or fossil fueled power generator to ensure supply security and reliability. Hybrid power systems consist of the integration of the generations of different natures for the use of power of an electrical nature. These hybrid power grids may be independent of a large area based and centralized nature based electric utility grid and incorporate power generation sources which have a different nature. These may have small size to large size islanded grids having size in the form of megawatts. Small size hybrid grids may include individual household power supply systems rated at one kilowatt which has the rooftop integrated solar PV plants. The hybrid power networks are capable of delivering currents of alternating nature which has a frequency of fixed nature. These have incorporated recent emerging technology which can be used to supply electric power in locations which are very remote. They have the advantages of easy transformation of AC power from one voltage level to another which minimizes loss in power when required to transfer over long distances. AC power systems when operated in isolated mode include the following essential components:

- Conventional AC diesel generators;
- Electrical distribution system network;
- AC loads distributed over the network.

**Figure 4:**
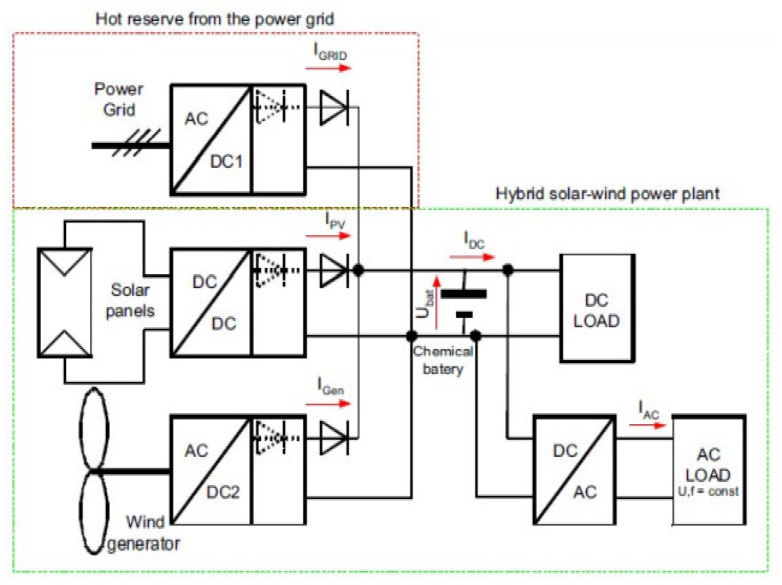
Block diagram of the hybrid solar–wind power plant.

However, the hybrid system may also incorporate the additional sources of power like RE sources including the wind energy, solar energy and storage. Storage may a source or a load. Systems of large size generally rated above 100 kW contain diesel generators, RE sources, loads, and storage of energy. For such system the storage needs to be of larger size because the renewable energy could be produced 24 × 7, which eventually suggests that the storage system should be capable to handle the continuous flow of energy. Moreover, sometimes the energy production could be high or sometimes low depending upon the natural conditions; for example, hydropower plants sometimes face higher productions of energy due to the strong flow of water caused by the natural factors such as rain, so storage needs to be larger. These systems with ratings below 100 kW have the combination of both AC and DC components which are in common for the purpose of storage of energy. DC parts may include diesel generators, storage for energy and RE sources. There is some small hybrid system which only operates on DC loads, generally lesser compared to 5 kW which are being used commercially on the remote location for the purpose of repeater stations of communication systems. Operations of the hybrid network and interaction between the different constituent components have been illustrated. Figure 4 describes the idea of a power-plant that is hybrid in nature, meaning it consists of various methods to produce energy by intermixing two types of power source, the wind and sunlight.

## 4. AI-Based DNN Architectures for WBANs on Sporadic Elder Movements

We use the Markov model based on AI-Based DNN Architectures [42]: our proposed model the Markov Wireless Channel [43] as a two-state mechanism with no overall loss, but with a loss of overall, that can be generalized to several states to clearly understand the problem and encourage our theoretical study. The channel has two states: positive and negative. After that, each sensor node is connected to the central node. The packet sent via the node will be successfully transmitted if the node channel is in good condition; otherwise, the packet transmission will fail if the node channel is in bad condition.

(4) $$\mathrm{Probability}=\left[\begin{array}{cc} 1-{\mathrm{probability}}_{\mathrm{PT}} & {\mathrm{probability}}_{\mathrm{LT}}\\ {\mathrm{probability}}_{\mathrm{LT}} & 1-{\mathrm{probability}}_{\mathrm{PT}} \end{array}\right]$$

In this case, LT is the likelihood of transformation from good to bad, and PT is the probability of transition from bad to good. The first vector of the condition of a connection is x (0) and the connection is in good condition with a 1 and a 0. The possibility that the channel is in good condition after the link has had time slots.

As seen, TL = probability PT + probability LT, and probability () is a monotonous function. If the initial condition is good, the function is a monotonous decrease with a low PPT/TL value; if the initial condition is worse, the function is a monotonous increase with a high probability PT/TL value. According to this formula, we can calculate the probability that the channel will be in good condition on each slot, as well as the likelihood that if the channel is in good condition, the transmission will succeed, so that the likelihood of good channel quality will equal the risk of data transmission success. The channel characteristics are then linked to the transmission success rate.

(5) $$\mathrm{Probability}\;(\tau )=[1-A\;(0)\;A\;(0)]\;{\left(\mathrm{probability}\right)}^{\tau }\left[\left(\begin{array}{c} 0\\ 1 \end{array}\right)\right]$$

$$\{\begin{array}{c} \frac{{\mathrm{probability}}_{\mathrm{PT}}}{Z}-\frac{{\mathrm{probability}}_{\mathrm{PT}\;}{\left(1-Z\right)}^{\tau }}{Z}\;\;A\left(0\right)=0\\ \frac{{\mathrm{probability}}_{\mathrm{BG}}}{Z}-\frac{{\mathrm{probability}}_{\mathrm{BG}\;}{\left(1-Z\right)}^{\tau }}{Z}\;\;A\left(0\right)=0 \end{array}$$

$$\{\begin{array}{c} {\mathrm{probability}}_{\mathrm{PT}}\\ Z \end{array}-\frac{{\mathrm{probability}}_{\mathrm{PT}\;}{\left(1-Z\right)}^{\tau }}{Z}$$

**Figure 5:**
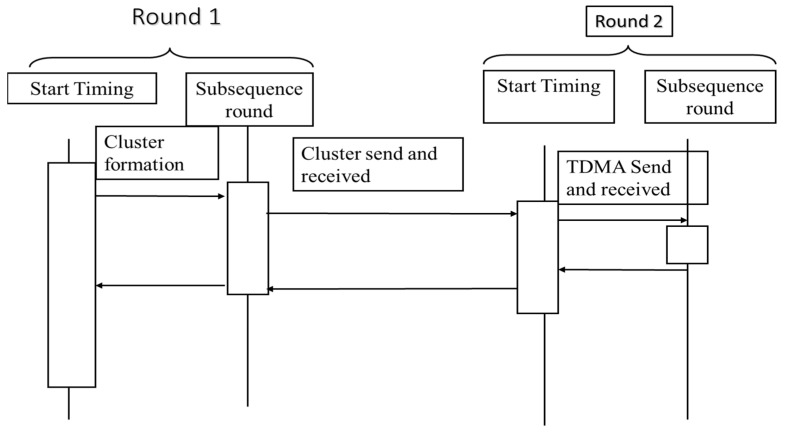
Intelligent Sleeping Mechanism (ISM).

Known as an “Intelligent Sleeping Mechanism,” it is designed to help you sleep better. In order to calculate an average value for each data packet, cluster heads collect packets from their cluster nodes and put them together. The initial data packet is sent to the base station by the cluster heads. This threshold is set by the user in compliance with the sensed software. For example, in agricultural land, it may be humidity; if this moisture drops below a certain threshold, it appears that the value in this data round will reach a critical value and remain stable for some time. In exchange, these nodes and their cluster heads send energy data to the base station, which ends its position throughout. The remaining clusters round off and submit their data in their set TDMA slots to the database station. AI-based DNN architectures for WBANs on Sporadic Elder Movements timeline [34], as stated, is shown in Figure 5.

## 5. Composition of the WSN

Energy consumption of sensor nodes [44]: In this section, to represent the, consumes the least amount of sleep energy. Therefore, contact in the network is more energy-efficient due to the unnecessary forwarding and acceptance and the acceleration of sleeping activation. Productive energy usage can help to extend the lives of the WSN.

Proposed Sleep Time optimization [45] in WBANs for energy efficiency optimization problem WBANs are distributed networks that must often coexist with one another. Each network has its transmission capacity, and technical cooperation among all WBANs to prevent co-channel interference is difficult to maintain. Since traditionally allocated transmission capacity is not optimized, there is a lot of space to optimize power in WBANs. These traditional algorithms can cause significant interference in nearby WBANs. To reduce gateway interference, we propose a new power algorithm. There are various WBANs in the vicinity of each gateway. The algorithm is based on convex optimization and the lighting problem. The interference limits, or zone perimeter, are divided into linear, equally spaced, small k patch different for calculating transmission power, the interference limits, or zone perimeter. Any patch’s interference must not surpass Ides, also known as the interference cap, ensuring that adjacent WBANs do not interfere shows the power level obtained in the center of each interference boundary patch:

(6) $${\mathrm{interference}\;\mathrm{boundary}\;\mathrm{patch}}_{k}=\sum_{j=1}^{m}a_{kjP_{T}},$$

where *m* is the transmitter number (base stations), since there is only one gateway by which the area is served, *m* = 1 and PT are the optimum transmitter capacity. *a_kj_* is the transmission loss from transmitter to path *k*, where the degree of interference is measured. The path loss and the channel model shown in section is used to measure *a_cj_*. The algorithm calculates the transmission power to meet this interference criterion for the given gateway since Ides are known (it is empirically determined). The objective function is formulated for optimization purposes in optimization purposes.

Minimize

(7) $$max_{k=1,\;\ldots \ldots ,\;n}\log_{I_{k}}-{\mathrm{logI}}_{des}\;0\leq {\mathrm{probability}}_{j}\leq {\mathrm{probability}}_{jmax,}$$

**Figure 6:**
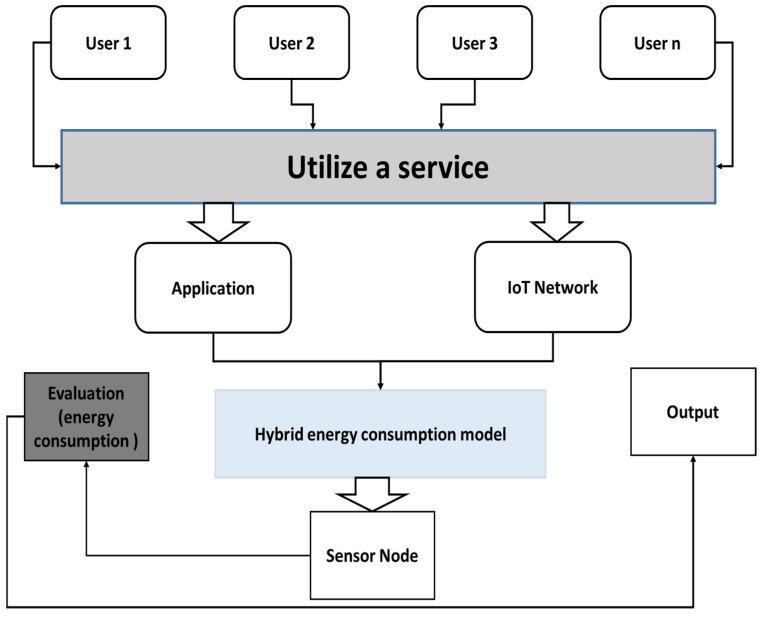
Sleep Time optimization in WBANs for energy efficiency.

The AI-Based DNN Architectures based RL (reinforcement learning [46]. In a discrete-time stochastic check, the overall RL problem is formalized. The following is how the agent interacts using his environment. the agent begins to obtain the initial observation—all 0—all in a given state in his environment s0 = all. The agent must take action at § A in any move t. As shown in Figure 6, three effects are: (i) the reward rt is received in the form of R; the transition state into st +1 (s); and (ii) the reward +1 in the form of an observation +1/s) is achieved. To suggested this controlled environment providing comprehensive treatment of RL fundamentals. Here, before examining the deep RL in the following research work, we study the key elements of RL.

AI-Based DNN Architectures [47], this property is known as the Markov property. Let’s start with Markovian stochastic control processes for the sake of simplicity.

**Figure 7:**
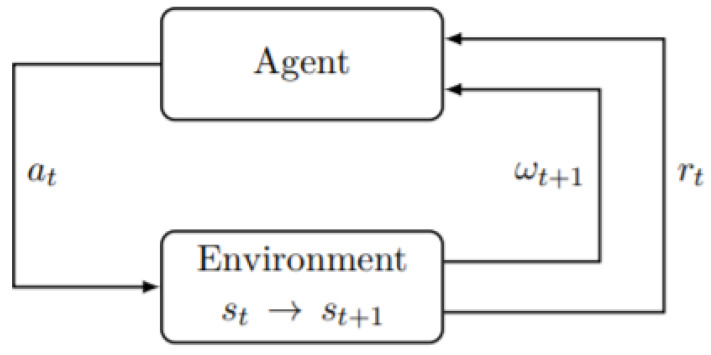
Agent and Environment.

Figure 7 shows the interaction between the Agent and the Environment to take an action depending on what is perceived from the Environment. This is one of the most popular and efficient methodology seen in AI based devices. Figure 7 describes how the changes in the environment are captured by the sensors and an appropriate signal is sent to the agent to take the respective action. The transition state of the environment is updated as soon as any change is detected. The signal is then sent to the agent along with a reward for successful detection of the change and taking action which enhances the overall performance of the model.

**Definition** **1.** *Markovian stochastic control is indeed a discrete-time stochastic process (i.e., it has the Markov property) if*

(8) $$P(\omega_{t+1}\;\omega_{t,a_{t}})\;=\;P(\omega_{t+1}\;\omega_{t,a_{t},\;\ldots \ldots ,\;}\omega_{0,}a_{0}),\;\mathrm{and}\;P(r_{t+1}\;\omega_{t,a_{t}})\;=\;P(r_{t}\;\omega_{t,a_{t},\;\ldots \ldots ,\;}\omega_{0,}a_{0}),$$

Authorities have little interest in looking backward in time because of Markov’s trait of just focusing on what is happening now.

## 6. Transmission Reliability

High redundancy is possible in the WSN for efficient data transfer. For e.g., the Arq retransmits data packets, and the FEC adds redundant packets to the source node. Furthermore, the stability of WSN data transmission is directly related to the performance of inter-nodal networking. One of the most common methods for enhancing data transfer efficiency is to lower channel loss rates by sending duplicate packets. It is a star network at its core, and the stability model varies depending on the requirements.

**Definition** **2.** *P is the average power consumption that represents the packet successfully transmitted [43]*

(9) $$E_{average}=\frac{Total\;energy\;consumed}{Number\;of\;packet\;received}$$

**Definition** **3.** *Represent the power consumed by the network coding. It is the normal process*

(10) $$\frac{E^{c}\;average}{E^{I}\;average}$$

## 7. Potential Improvements

Although the proposed model is optimal as per the terms of energy optimization, still there are various algorithms that could be implemented in the system to improve its performance. ANN is one of the most powerful algorithms of deep learning that is capable to make intelligent decision making model. It would work optimal for classification problems. Another technology that could be used is Deep learned recurrent type-3 fuzzy system. Fuzzy logic is an extension of Boolean logic. Not all events could be stated as true or false; some could be partially true or partially false. For such cases, designed fuzzy logic is used to improved the model. The deep learned recurrent type 3 fuzzy system is one of the best techniques for the models that are based on renewable energy. This system could be the substitute for our proposed technique.

Not all models could be fully optimized and so their optimization would be fractional, meaning that it would be between the range of 0 to 1, where 0 represents the worst efficiency and 1 represents best efficiency, and so the more the efficiency closer to 1, the better the model is. The research encourages the readers to implement the type-3 fuzzy logic systems in order to fill the gaps of the research. Deep learning is a complex yet most powerful existing concept or technology or algorithm. Using deep learning algorithms like recurrent type-3 fuzzy system would surely fill the gaps and improve the model to the next level.

Most of the research did not focus on the conservation of renewable energy and optimizing sensor energy consumption. Their main focus was to apply renewable energy to sensors. Some research did present excellent models that utilize minimal overall power but our research understood the main source for energy wastage and tried to optimize it.

## 8. Results Analysis and Discussion

This research focused on the power consumption of the model, then the research proposed by the AI based DNN architecture. Before we develop an energy saving model, we need to understand the power consumption of the model and which parts need the protocols to perform energy conservation. After discussing the power consumption, the study then proposed an AI-based DNN architecture. The reason behind using the proposed model is that the model would show intelligent behavior and the decisions taken would be optimal, which would eventually make the model energy conservative.

Our WBAN network using 48 source nodes and one coordinator was designed and simulated. The chest, belly, and arms all have nodes. Each node is at a permitted propagation distance from the sink node; moreover, the distances between the coordinator and the nodes are not the same. We used the Network OMNET++ environment to test our proposed WBAN protocol. Each source node receives 50 J of energy, except for the sink node, which receives 100 J for its numerous operations. Even though the allowed WBAN data rate ranges from 10 Kbps to 10 Mbps, the packet generation rate.

OMNET++ is one of the most popular and powerful simulation frameworks. As the research is based on wireless-ness and the network of sensors, the OMNET++ is one of the best tools that could be implemented in order to achieve an optimal result. The OMNET++ would build network simulators in order to build a stronger network between the sensors, which is wireless. The sensors network would be handled and simulated by the OMNET++ which would help the model to drain less energy and provide with an optimal network for the model.

Application for renewable energy modeling is a must to discuss as it is the result of the research. Most of the renewable energy using models is not monitored because of the abundance of the energy but it is crucial to optimize the energy to improve the efficiency of the model and so the research suggests this improved mode. Moreover, the renewable energy modelling would also describe how the model would perform in the long run.

In contrast to the literature the new protocols and data transfer reliability are assessed. From the single hop point, various literature techniques tailor the transmission to a reliable transmission. WBAN configurations that are popular in the industry are used. PLR reduction is implemented as a redundancy measure, i.e., packet loss rate gain by sending the same data piece into the same transmission region using TDMA’s packet loss rate strategy. At random between 0 and 1, with the exception that a value of 1 (i.e., r1 + r2 + r3 = 1) must be chosen. The selected parameters as modifications (r1: energy ratio, r2: connection reliability, and r3: SAR) have been thought distinct (keeping other parameters constant). In the application of individual metrics, the response of these measures varies from one to the other, with some indicators outperforming the others. The simulation results show that many parameters in the cost function must be included to meet such compromises. The proposed model is efficiency and provides the best energy savings results and maximizes long life. The QoS enhancement combines parametric cost function variations with a tweak to the minimum contest.

Related studies have mainly focused on the renewable energy utilization but not on the optimization of sensor energy consumption. Their aim was to apply renewable energy to various models. Those studies presented excellent models that utilized minimal overall energy, but our research found the main source for energy wastage and suggested to optimize it. Some papers discussed the optimization of rotor speed to improve overall efficiency, but as we are aware that not all models would have rotors, this model would not be applicable to a majority of the models. Our research aims to optimize the sensors because the world is changing to advanced production with the revolution of digital and wireless technology, so sensors would be a part of almost every model and so optimizing it would be better.

### The Required Evaluation Metrics for Healthcare Applications

Network topology, postural rotation, diminished resources, operation reliability calculations, radiation and disruption, global network life, and heterogeneous conditions are all examples of routing issues. Considering both of these factors, the essential performance metrics to be considered can be inferred and identified in the WBAN implementation process. The dimensions are described in the following section:

As nodes are 48, the average CSMA/CA delay increases to 62 ms, but Burst Bandwidth will shorten the end-to-end delay to 22 ms. We allow Burst Bandwidth allocation in the simulation when the average node latency is more significant than 15 ms. Any node will request Burst Bandwidth during the GTS (Geologic Time Scale) interval where there are no collisions.

When the number of nodes exceeds 18, the mean end-to-end delay is minimized using Burst Bandwidth. The slight drop in the end-to-end delay is an artifact of short simulator cycles. In IEEE 802.15.4, GTS is less effective than the BodyMAC system in bandwidth consumption, particularly as it becomes more complex. BodyMAC has improved performance in the GTS plus Burst Bandwidth. The below graphs depicts how end to end delay caused the results to be good or bad in terms of energy efficiency. The following graphs are really important to understand how our proposed model is better than the existing method and how it performs for networks and nodes. The graphical representation is really important to understand why we need to improve the existing methodology because of its dull end to end delay. The three factors that would be used to show the need for energy efficiency are the number of nodes, mean packet arrival time, and the time. All three of these factors would be judged with end-to-end delay to show how the existing method performs for these features and how our proposed method performs for the same three features. From the result, we could say that the performance of the proposed method is better than the existing method.

**Figure 8:**
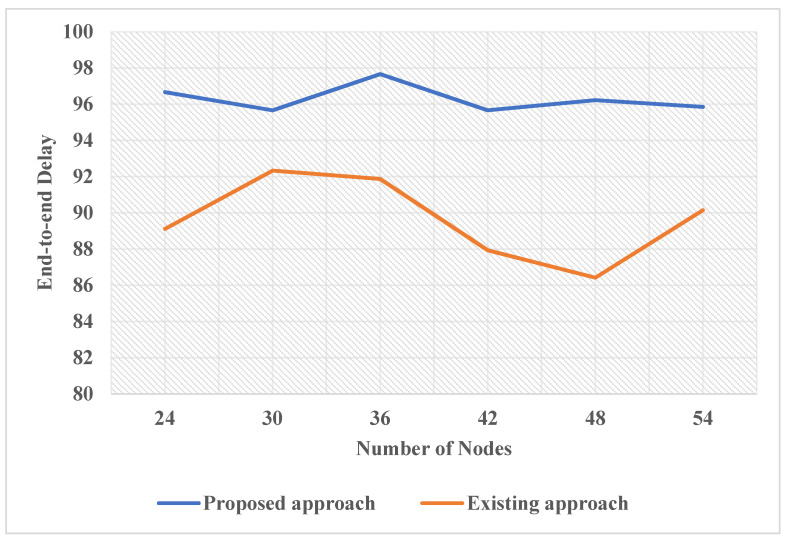
Comparative Analysis in term of End-to-end delay, number of nodes utilization of energy consumption.

Figure 8 describes the change in End-to-End delay as the number of nodes of the existing approach and the proposed approach increases. As it is shown, the end-to-end delay in the existing approach is overall less as the number of nodes increases than the proposed approach, which makes the proposed approach more efficient. Along with that, the proposed approach shows consistency as the number of nodes increases. The curve is consistent and not very variable in the case of the proposed approach, whereas for the existing approach, the curve is inconsistent as it goes up and down for an increasing number of nodes, which is unstable. Therefore, the proposed approach performs better and is more stable, consistent and efficient than the existing approach.

**Figure 9:**
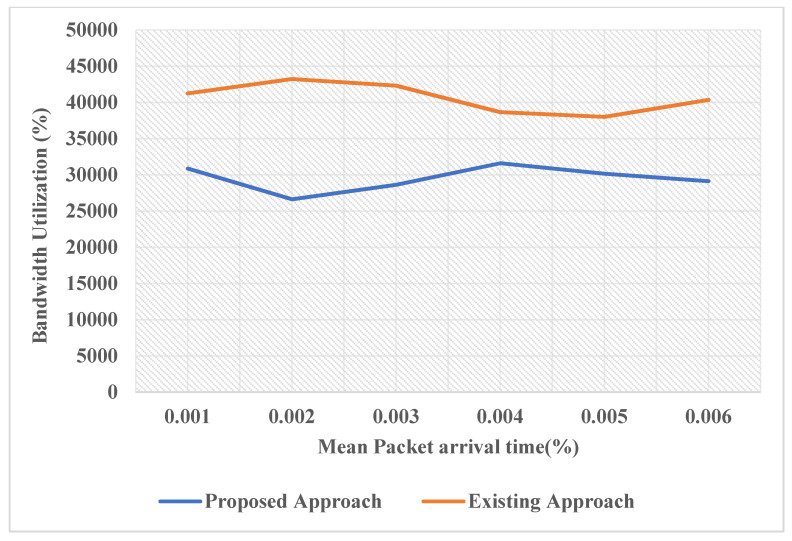
Comparative Analysis between bandwidth utilization and means packet arrival time.

**Figure 10:**
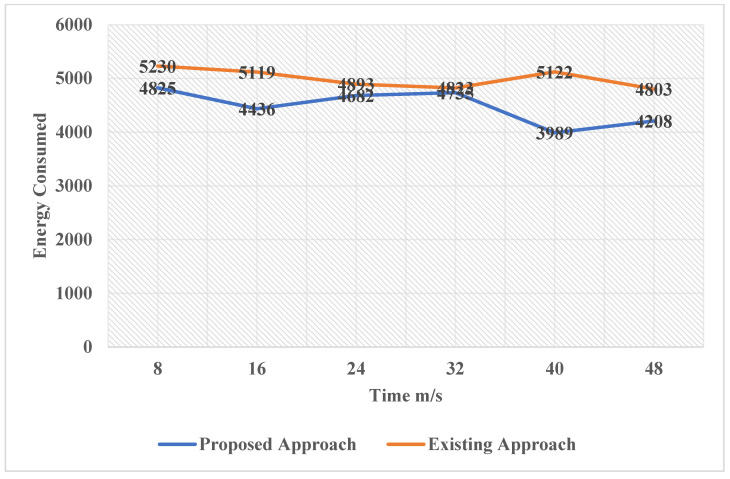
Energy Consumed by each Node as a Function of Time.

Although it is important to understand that just the basics algorithms of AI will not sufficient make the machine intelligent to be considered advanced deep learning algorithm. The deep learning, which is a subset of machine learning, is one of the most powerful domains that is capable of developing machines intelligent enough to make our own decisions and learn with time. The future research could implement the ANN (Artificial Neural Network), which is inspired from the biological neural network, to improve the performance of the system and make it truly intelligent. After implementing the deep learning in the model, its application domain would expand as its capability would increase. After discussing the above graphs, now the study can conclude that the proposed model is an intelligent one, unlike its previous models. In Figure 9, it shows comparative analysis between bandwidth utilization and means packet arrival time. The proposed model makes intelligent decisions by detecting the excess energy consumption factors and eliminating them to undertake energy optimization. In Figure 10, it shows the energy consumed by each node as a function of time.

## 9. Conclusions

This paper demonstrated how WBANs are used as a core infrastructure for continuous and continuing ambulatory health monitoring. These new developments have the potential to benefit patients, medical practitioners, and the environment by providing continuous ambulatory monitoring, performance in terms of energy efficiency of Sporadic Elder Movements (SEM) during various daily operations, and controlled rehabilitation, as well as possible intelligence discovery and data processing of all data gathered. Motion sensors are specified in the general Wi-Fi architecture, which is essential for its implementation. We also addressed many critical technical problems, such as sensor node hardware configuration and energy conservation. Using graphical representation, the study concluded that the proposed model is an intelligent and the algorithm is able to make intelligent decisions. The proposed model makes intelligent decisions by detecting the excess energy consumption factors and eliminating them to do energy optimization. Additional measures are needed to increase wireless networking efficiency, sensor node reliability, protocol security, and interoperability standardization.

The model focuses on the sensor energy optimization, which is one of the most vital components in the model. Implementing the discussed methodology would improve its performance which would eventually improve the performance of the system. The research not only focuses on the small point but also on social factors. It encourages energy efficiency of any kind and promotes the overall development of the system. However, it does require some future work, the world of technology is evolving quickly, new and powerful algorithms are taking over the weaker ones, so those powerful algorithms could be implemented on the system to keep it up to date, as with fuzzy logic. Our proposed AI-based DNN could be very effective for Sensor Energy Optimization, as shown in the results, as long as the correct conditions are provided and all the steps are performed correctly. This research is helpful for the concept of energy efficiency and it also promotes the idea to conserve the energy as much as we can. This study aims to promote energy conservation in the society for different products and models. Some of the devices, technologies and models are working on energy far more than they actually need because of technical issues or less efficiency of the model, this study suggests and promotes the need to be energy efficient models to enhance the working of the system.

## Figures and Tables

**Table 1:** Energy consumption protocol in wireless body area networks.

S. No.	Study	Machine Learning/Deep Learning Technique	Application	Research Gaps
1.	Amir, M.F. et al., 2018 [21]	Deep Neural Network (DNN)	Neurosensor-a CMOS image sensor	Static Random Access Memory (SRAM) configuration are not included.
2.	Yosuf, B.A. et al., 2021 [22]	Deep Neural Network (DNN)	To analyse data over Virtualized Cloud Fog Network (CFN).	CFN architectures time frames were not explained.
3.	Wang, P. et al., 2021 [23]	Deep Neural Networks(DNN)	Predicting the performance of thermoelectric generators (TEGs)	The minimum and maximum frequency were not known.
4.	Nguyen, C.T. et al., 2020 [24]	Artificial Neural Networks (ANN)	Emerging technologies in the sensors required for social distancing.	Context of 6G technology and blockchain technologies has not been explained.
5.	Mrabet, H. et al., 2020 [25]	Deep Belief Network (DBN)	Analysis of IoT based security layers in sensors.	Dependency of different layers on sensor is illuminated.

**Table 2:** Comparative study for analysis of power optimization for low-energy based sensor used in wireless sensor networks.

Study	Primary Purpose of Study	Methodology Used
Khan M.J., et al., 2020 [26]	To increase the efficiency of rotor speed for generation of wind energy.	Optimal torque control method’s (OPT) efficiency is better than other methods.
Xu, W. et al., 2015 [27]	To elevate the levels of energy optimization for Wireless Sensor Networks (WSN) where energy level is highly unstable.	Lyapunov drift-plus-penalty with perturbation technique is applied to obtain more persistent results.
Qi, N. et al., 2021 [28]	To propose scheme of hybrid-diode topology for Solar energy based WSN.	To optimize the energy levels, one-port hybrid diode topology is used.
Periola, A.A. et al., 2021 [29]	To tackle the major issues for developing mobile wind turbine systems.	The sensor readings for wind speed are collected using drone-based networking units.
Shakeel, M. et al., 2021 [30]	To build a system for low level energy harvesting.	Lyapunov drift-plus-penalty with perturbation technique is applied to obtain more persistent results.

**Table 3:** Sensor in WBAN.

Wearable Sensors	Implatable Sensors
EEG	Retina Implants
Glucose Sensor	Deep brain stimulator
EMG	Electronic Pill
ECG	Pace maker
Blood Presure	Electronic Pill for drug delivery

## Data Availability

Data will be shared for review based on the editorial reviewer’s request.
